# Procedural outcome & risk prediction in young patients undergoing transvenous lead extraction—a GALLERY subgroup analysis

**DOI:** 10.3389/fcvm.2023.1251055

**Published:** 2023-09-06

**Authors:** Enida Rexha, Da-Un Chung, Heiko Burger, Naser Ghaffari, Tomas Madej, Virgilijus Ziaukas, Kambiz Hassan, Hermann Reichenspurner, Nele Gessler, Stephan Willems, Christian Butter, Simon Pecha, Samer Hakmi

**Affiliations:** ^1^ Department of Cardiology and Critical Care Medicine, Asklepios Klinik St. Georg, Hamburg, Germany; ^2^ Department of Cardiac Surgery, Kerckhoff Klinik, Bad Nauheim, Germany; ^3^ Department of Cardiovascular Surgery, Helios Clinic for Heart Surgery, Karlsruhe, Germany; ^4^ Department of Cardiac Surgery, University Heart Center Dresden, Dresden, Germany; ^5^ Department of Cardiac Surgery, Schüchtermann-Klinik, Bad Rothenfelde, Germany; ^6^ Department of Cardiac Surgery, Asklepios Klinik St. Georg, Hamburg, Germany; ^7^ Department of Cardiovascular Surgery, University Medical Center Hamburg-Eppendorf, Hamburg, Germany; ^8^ DZHK (German Centre for Cardiovascular Research), Partner Site Hamburg/Kiel/Luebeck, Hamburg, Germany; ^9^ Department of Cardiology, Heart Center Brandenburg Bernau, Neuruppin, Germany

**Keywords:** transvenous lead extraction, cardiac implantable electronic device, lead management, young adults, outcomes, risk factors

## Abstract

**Background:**

The prevalence of young patients with cardiac implantable electronic devices (CIED) is steadily increasing, accompanied by a rise in the occurrence of complications related to CIEDs. Consequently, transvenous lead extraction (TLE) has become a crucial treatment approach for such individuals.

**Objective:**

The purpose of this study was to examine the characteristics and procedural outcomes of young patients who undergo TLE, with a specific focus on identifying independent risk factors associated with adverse events.

**Methods:**

All patients in the GALLERY (GermAn Laser Lead Extraction RegistrY) were categorized into two groups based on their age at the time of enrollment: 45 years or younger, and over 45 years. A subgroup analysis was conducted specifically for the younger population. In this analysis, predictor variables for all-cause mortality, procedural complications, and procedural failure were evaluated using multivariable analyses.

**Results:**

We identified 160 patients aged 45 years or younger with a mean age of 35.3 ± 7.6 years and 42.5% (*n* = 68) female patients. Leading extraction indication was lead dysfunction in 51.3% of cases, followed by local infections in 20.6% and systemic infections in 16.9%. The most common device to be extracted were implantable cardioverter-defibrillators (ICD) with 52.5%. Mean number of leads per patient was 2.2 ± 1.0. Median age of the oldest indwelling lead was 91.5 [54.75–137.5] months. Overall complication rate was 3.8% with 1.9% minor and 1.9% major complications. Complete procedural success was achieved in 90.6% of cases. Clinical procedural success rate was 98.1%. Procedure-related mortality was 0.0%. The all-cause in-hospital mortality rate was 2.5%, with septic shock identified as the primary cause of mortality. Multivariable analysis revealed CKD (OR: 19.0; 95% CI: 1.84–194.9; *p* = 0.018) and systemic infection (OR: 12.7; 95% CI: 1.14–142.8; *p* = 0.039) as independent predictor for all-cause mortality. Lead age ≥ 10 years (OR: 14.58, 95% CI: 1.36–156.2; *p* = 0.027) was identified as sole independent risk factor for procedural complication.

**Conclusion:**

TLE in young patients is safe and effective with a procedure-related mortality rate of 0.0%. CKD and systemic infection are predictors for all-cause mortality, whereas lead age ≥ 10 years was identified as independent risk factor for procedural complications in young patients undergoing TLE.

## 1. Introduction

The prevalence of cardiac implantable electronic devices (CIED) is continuously increasing, with a high number of such devices present in young adults (1, 2). Along with this development there is a concomitant increase in the incidence of CIED-related complications, such as device-related infections (CDRI) and lead failure (3–7). Multiple pulse generator replacements, device revisions and an increase in device complexity are associated with a higher lifetime risk for CIED-related complications (7, 8). Transvenous lead extraction (TLE) has evolved into an indispensable therapeutic option for such cases. According to the international consensus statements in patients with CDRI a complete lead extraction should be aimed at and abandoned leads should be avoided (9, 10). However, there are no clear recommendations regarding lead extractions for non-infectious indications.

Abandoned leads contribute to a greater number of existing leads, leading to challenges in venous access and potential damage to the tricuspid valve (11–13). Moreover, the presence of abandoned leads worsens the overall clinical outcome of lead extraction procedures, primarily due to their prolonged lead dwell time (14).

Additionally, the young age of patients at the time of their initial implantation, regardless of the duration of the dwell lead time, is correlated with a higher occurrence of increased connective tissue proliferation on the leads and the formation of adhesions between the leads and the cardiac structures (15–17). Consequently, this results in poorer therapeutic TLE outcomes in this particular group of patients (15).

On the other hand, recent extensive registries have demonstrated that performing TLE procedures in experienced centers is a safe and effective approach (6, 13, 18).

The objective of this study was to perform a subgroup analysis of the GermAn Laser Lead Extraction RegistrY (GALLERY) to examine the patient characteristics and procedural outcomes specifically for young patients who undergo TLE. Furthermore, the study aimed to identify independent risk factors associated with adverse events in this particular subgroup.

## 2. Methods

### 2.1. Patients population & study design

The GALLERY included all patients who underwent laser lead extraction (LLE) at 24 participating centers during the period from January 2013 to March 2017. The registry enrolled a total of 2,524 patients with a total of 6,117 leads treated.

This study is a post-hoc subgroup analysis that specifically focuses on patients who were 45 years old or younger at the time of enrollment (Group A) within the larger GALLERY study. For comparison, patients who were over 45 years old were designated as the comparative group (Group B). The data collection methods and study design have already been published in detail in the GALLERY main manuscript (6). The study adheres to the principles outlined in the Declaration of Helsinki, and the study protocol received approval from the ethics committee of the state medical board Hamburg (reference number: WF-026/17).

### 2.2. Definitions

All definitions pertaining TLE procedure, tools and techniques, as well as procedural complications and outcomes, adhere to the specifications published by the HRS and EHRA expert consensus statements (9, 10). Complete lead removal was defined as the extraction of all targeted lead material from vascular space. Incomplete lead removal was defined as remaining leads or fragments (>4 cm) in the patient's body by the end of the procedure. Complete procedural success was determined by removal of all targeted leads in absence of any permanently disabling complication or procedure-related death. Clinical procedural success was defined as retention of small lead fragments that do not negatively impact the goals of the procedure, in absence of any permanently disabling complication or procedure-related death. Procedural failure was defined as the inability to achieve complete procedural- or clinical success, and/or any permanently disabling complication or procedure-related death. Major complications were defined as complications which were either life-threatening or resulted in death or any other significant persisting disabling condition. A minor complication was every procedure-related undesired event, leading to a medical or minor procedural intervention without persistent influence on patient's functional capacity. Procedure-related death was defined as any death that occurred during the extraction procedure or was directly or indirectly associated with a procedural complication. In-hospital mortality was defined as any death (cardiac or non-cardiac) that occurred during the hospital stay, irrespective of its relation to the procedure.

### 2.3. Lead extraction management

All procedures were performed in an operating room under general anesthesia, guided by fluoroscopy. Prior to the procedure, all patients were prepared for the possibility of emergent sternotomy, with a cardiopulmonary bypass circuit ready for immediate use if required. In two of the participating centers the primary extractor was an electrophysiologist, while in the other centers, cardiac surgeons were primarily involved in the lead extraction procedures. In most cases, a heart team, consisting of both electrophysiologists and cardiac surgeons, worked together to perform the extractions. Leads were dissected from the surrounding tissue and the sleeves were removed. Lead locking devices (LLD) were then inserted. LLE was performed using either the Glide-Light 80 Hz or SLS II 40 Hz laser sheaths. The sheath sizes utilized ranged from 14 to 16 French.

If necessary, the use of additional powered or non-powered extraction sheaths such as mechanical rotational sheaths, non-powered extraction sheaths, snares, or other tools was permitted, as long as at least one laser sheath was utilized during the extraction procedure. After the removal of the extraction sheath, the subclavian access site was sutured to ensure proper hemostasis and prevent potential complications.

### 2.4. Study objective

The primary objective of the study was to analyze the characteristics and procedural outcomes of all patients who underwent LLE and were 45 years old or younger (Group A). This group was compared to patients who were older than 45 years (Group B). The focus was on examining patient characteristics and evaluating the procedural outcomes in both age groups. Additionally, the study aimed to identify independent risk factors that could predict adverse events.

### 2.5. Statistical analysis

Continuous variables are expressed as mean ± standard deviation (SD) for normal distributions and median and interquartile range (IQR) for non-gaussian distributions. Categorial variables are shown as counts and percentages. Categorical variables between groups were compared using *χ*^2^-test or Fisher's exact test, in case of small sample sizes (<5 counts per cell). Continuous variables between 2 groups were compared with Mann–Whitney–*U* test. Continuous variables between >2 groups were compared using the Kruskal–Wallis test. Univariable and multivariable logistic regression analysis was used to determine independent predictors for all-cause in-hospital mortality, procedural complications and -failure. Predictor variables that reached statistical significance in univariable analysis and additional clinically relevant covariables were included into the multivariable analyses. A 2-tailed *p*-value of <0.05 was considered as statistically significant. Statistical analysis was performed using Prism 8 (GraphPad Software, San Diego, CA, USA) and IBM SPSS 25.0 statistical software package (IBM, Armonk, NY, USA).

## 3. Results

### 3.1. Patient characteristics

We identified 160 patients (6.3% of the GALLERY) of age 45 years or younger with a mean age of 35.3 ± 7.6 years (Group A). The mean age in Group B was 70.3 ± 11.0 with a significant difference between both groups (*p* < 0.001). The number of female patients and patients presenting with sinus rhythm at admission was significantly higher in Group A (*p* < 0.001).

In Group B, arterial hypertension (AHT) was present in 73.6% of patients, coronary artery disease (CAD) in 45.3%, diabetes mellitus (DM) in 32.9%, and chronic kidney disease (CKD) in 32.9%. These rates were found to be significantly higher compared to Group A (*p* < 0.001 for all comparisons). Further patient characteristics are summarized in Table 1.

**Table 1:** Patient demographics and clinical characteristics between groups.

	Group A (*n* = 160)	Group B (*n* = 2,364)	*p*-value
Mean age, years ± SD	35.3 ± 7.6	70.3 ± 11.0	<0.001
Female sex, *n* (%)	68 (42.5)	563 (23.8)	<0.001
Mean BMI, kg/m^2^ ± SD	25.6 ± 5.4	27.2 ± 4.5	<0.001
LVEF ≤ 30%, *n* (%)	18 (11.3)	639 (27.0)	<0.001
Arterial hypertension, *n* (%)	38 (23.8)	1,741 (73.6)	<0.001
Coronary artery disease, *n* (%)	12 (7.5)	1,072 (45.3)	<0.001
Diabetes mellitus, *n* (%)	10 (6.3)	778 (32.9)	<0.001
Chronic kidney disease, *n* (%)	8 (5.0)	777 (32.9)	<0.001
Previous heart surgery, *n* (%)	28 (17.5)	584 (24.7)	0.049
Pacemaker dependency, *n* (%)	49 (30.6)	754 (31.9)	0.806
ECG on admission			
– Sinus rhythm, *n* (%)	105 (65.6)	1,198 (50.7)	<0.001
– Atrial fibrillation, *n* (%)	3 (1.9)	507 (21.4)	<0.001
– Paced rhythm, *n* (%)	52 (32.5)	659 (27.9)	0.243
Extraction indications			
Local infection, *n* (%)	33 (20.6)	855 (36.2)	<0.001
Systemic infection, *n* (%)	27 (16.9)	695 (29.4)	0.001
Lead dysfunction, *n* (%)	82 (51.3)	694 (29.4)	<0.001
Upgrade, *n* (%)	4 (2.5)	52 (2.2)	0.978
Vascular pathology, *n* (%)	1 (0.6)	16 (0.7)	0.673
Chronic pain, *n* (%)	5 (3.1)	11 (0.5)	<0.001
Other, *n* (%)	8 (5.0)	41 (1.7)	0.009

Values are expressed as mean ± SD or counts (*n*) and percentages (%), *p*-values < 0.05 were considered statistically significant; BMI, body mass index; ECG, electrocardiogram; Group A: age ≤ 45 years, Group B: age > 45 years; LVEF, left ventricular ejection fraction; SD, standard deviation.

In Group A, the most frequent indication for lead extraction was lead dysfunction, accounting for 51.3% of cases which was significantly more prevalent compared to Group B (51.3% vs. 29.4%, *p* < 0.001). In Group B the most common indication for lead extraction was local infection, followed by systemic infection, whose rates were both significantly higher compared to Group A (36.2% vs. 20.6%, *p* < 0.001 and 29.4% vs. 16.9%, *p* = 0.001, respectively). In Group A, more patients underwent TLE due to device-related pain compared to Group B (3.1% vs. 0.5%, *p* < 0.001).

**Figure 1:**
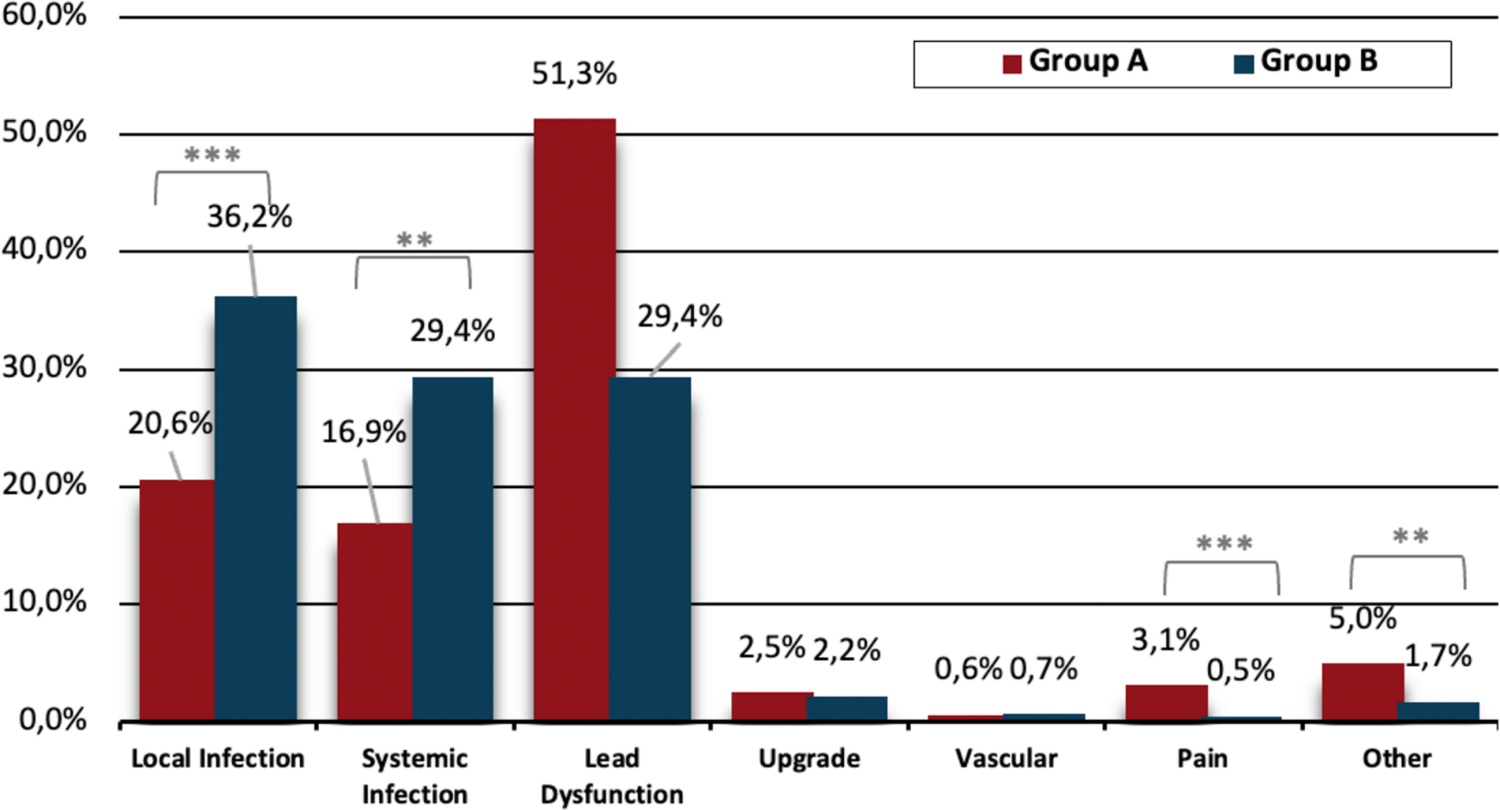
Extraction indication between groups. Group A: Age ≤ 45 years, Group B: Age >45 years, **p<0.01, ***p<0.001.

The extraction indications are shown in Figure 1.

### 3.2. Device and lead characteristics

There was no significant difference in the total number of patients with pacemakers (PM) between the two groups (36.3% vs. 41.1% *p* = 0.263). In Group A, the most extracted devices were implantable cardioverter-defibrillators (ICDs), accounting for 52.5% of the cases. This prevalence was significantly higher compared to Group B (52.5% vs. 32.6% *p* < 0.001). Conversely, cardiac resynchronization therapy (CRT) devices were significantly more prevalent in Group B (26.4% vs. 11.3%, *p* < 0.001). The mean number of total leads was significant higher in Group B (2.4 ± 1.0 vs. 2.2 ± 1.0, *p* = 0.002). There were no significant differences between groups in terms of the median age of the oldest lead and the number of patients with abandoned leads (*p* = 0.174 and *p* = 0.353). Further device and leads characteristics are shown in Table 2.

**Table 2:** Device & lead characteristics.

	Group A (*n* = 160)	Group B (*n* = 2,364)	*p*-value
– Pacemaker, *n* (%)	58 (36.3)	971 (41.1)	0.263
– ICD, *n* (%)	84 (52.5)	770 (32.6)	<0.001
– CRT, *n* (%)	18 (11.3)	625 (26.4)	<0.001
Total number of leads, *n*	348	5,769	
Mean number of total leads, *n* ± SD	2.2 ± 1.0	2.4 ± 1.0	0.002
Dual-coil ICD leads, *n* (% of total leads)	44 (12.6)	963 (16.7)	0.057
Lead fixation mechanism			
Active fixation, *n* (% of total leads)	228 (65.5)	3,746 (64.9)	0.870
Passive fixation, *n* (% of total leads)	71 (20.4)	1,467 (25.4)	0.04
Unknown fixation, *n* (% of total leads)	49 (14.1)	556 (9.6)	
Median age of oldest lead, months [IQR]	91.5 [54.75–137.5]	96 [62; 141]	0.174
Number of patients with right sided leads, *n* (%)	45 (28.1)	791 (33.5)	0.193
Number of patients with abandoned leads, *n* (%)	44 (27.5)	741 (31.4)	0.353

Values are expressed as mean ± SD, median with IQR or counts (*n*) and percentages (%), *p*-values < 0.05 were considered statistically significant; CRT, cardiac resynchronization therapy; Group A: age ≤ 45 years, Group B: age > 45 years, ICD, implantable cardioverter-defibrillator; IQR, interquartile range; SD, standard deviation.

### 3.3. Procedural data outcome

Median procedural time in Group A was 88 [65; 151.75] minutes and thus significantly longer than in Group B with 81 [55; 125] minutes, (*p* = 0.02).

The median duration of overall hospital stay, duration from admission to procedure and median postoperative stay was significantly shorter in Group A [6 vs. 9 (days), *p* < 0.001; 1 vs. 2 (days), *p* = 0.01; 4 vs. 6 days, *p* < 0.001, respectively].

Overall complication rate in Group A was 3.8% with 1.9% minor and 1.9% major complications and did not significantly differ compared to Group B (3.8% vs. 4.4%, *p* = 0.869; 1.9% vs. 2.3%, *p* = 0.950; 1.9% vs. 2.1%, *p* = 0.907, respectively).

Additional extraction tools such as mechanical rotating dilator (MRD) sheaths, snares, a combination of both, or other tools were utilized in a total of 8.8% of the patients in Group A and in 6.5% of the patients in Group B with no significant difference between the two groups (*p* = 0.253).

No significant difference was seen in complete procedural success (90.6% vs. 91.5%, *p* = 0.814), clinical procedural success (98.1% vs. 97.8%, *p* = 0.994) and procedure-related mortality (0.0% vs. 0.6%, *p* = 0.632). All-cause in-hospital mortality also did not show a significant difference between the groups (2.5% vs. 3.6%, *p* = 0.596) and was mainly due to septic shock in both groups. Details of procedural data are shown in Table 3.

**Table 3:** Procedural data.

	Group A (*n* = 160)	Group B (*n* = 2,364)	*p*-value
Median hospital stay, days [IQR]	6 [4; 11]	9 [6; 17]	<0.001
Median duration from admission to procedure, days [IQR]	1 [1; 3]	2 [1; 4]	0.01
Median postoperative stay, days [IQR]	4 [2; 7.75]	6 [3; 13]	<0.001
Median procedural time, minutes [IQR]	88 [65–151.75]	81 [55; 125]	0.02
Use of additional tools, *n* (%)	14 (8.8)	154 (6.5)	0.253
– Mechanical rotating dilator sheath (MRD)	10 (6.3)	100 (4.2)	0.312
– Snare	3 (1.9)	29 (1.2)	0.731
– MRD + Snare	1 (0.6)	17 (0.7)	0.891
– Other	–	8 (0.3)	
Overall complications, *n* (%)	6 (3.8)	103 (4.4)	0.869
Minor complications, *n* (%)	3 (1.9)	54 (2.3)	0.950
– Pocket hematoma, *n* (%)	2 (1.3)	45 (1.9)	0.772
– Pericardial effusion without intervention, *n* (%)	–	4 (0.2)	
– Pneumothorax, *n* (%)	1 (0.6)	3 (0.1)	0.613
– Subclavian vein thrombosis, *n* (%)	–	1 (0.04)	
– Pulmonary embolism, *n* (%)	–	1 (0.04)	
Major complications, *n* (%)	3 (1.9)	49 (2.1)	0.907
– Laceration of SVC or cavo-atrial junction, *n* (%)	1 (0.6)	25 (1.1)	0.905
– RA/RV perforation, *n* (%)	1 (0.6)	19 (0.8)	0.805
– Pericardial tamponade, *n* (%)	1 (0.6)	3 (0.1)	0.613
– Hemothorax, *n* (%)	–	2 (0.08)	
Complete procedural success, *n* (%)	145 (90.6)	2,163 (91.5)	0.814
Clinical success, *n* (%)	157 (98.1)	2,312 (97.8)	0.994
Procedure-related mortality, *n* (%)	0 (0.0)	15 (0.6)	0.632
All-cause mortality, *n* (%)	4 (2.5)	86 (3.6)	0.596
– Septic shock, *n* (%)	3 (1.9)	50 (2.1)	0.838
– Multiorgan failure (not further specified), *n* (%)	1 (0.6)	13 (0.5)	0.902
– Cardiogenic shock, *n* (%)	–	10 (0.4)	
– Hemorrhagic shock, *n* (%)	–	3 (0.1)	
– Mesenteric ischemia, *n* (%)	–	3 (0.1)	
– Fatal arrhythmia (VT/VF), *n* (%)	–	1 (0.04)	
– Asystole, *n* (%)	–	1 (0.04)	
– Pericardial tamponade, *n* (%)	–	1 (0.04)	
– Fatal pulmonary embolism, *n* (%)	–	1 (0.04)	
– Respiratory failure, *n* (%)	–	1 (0.04)	
– Acute renal failure, *n* (%)	–	1 (0.04)	
– Cerebrovascular accident, *n* (%)	–	1 (0.04)	

Values are expressed as median with IQR or counts (*n*) and percentages (%), *p*-values < 0.05 were considered statistically significant; Group A: age ≤ 45 years, Group B: age > 45 years, IQR, interquartile range; RA, right atrium; RV, right ventricle; SVC, superior vena cava; VF, ventricular fibrillation; VT, ventricular tachycardia.

### 3.4. Predictors for adverse events

**Figure 2:**
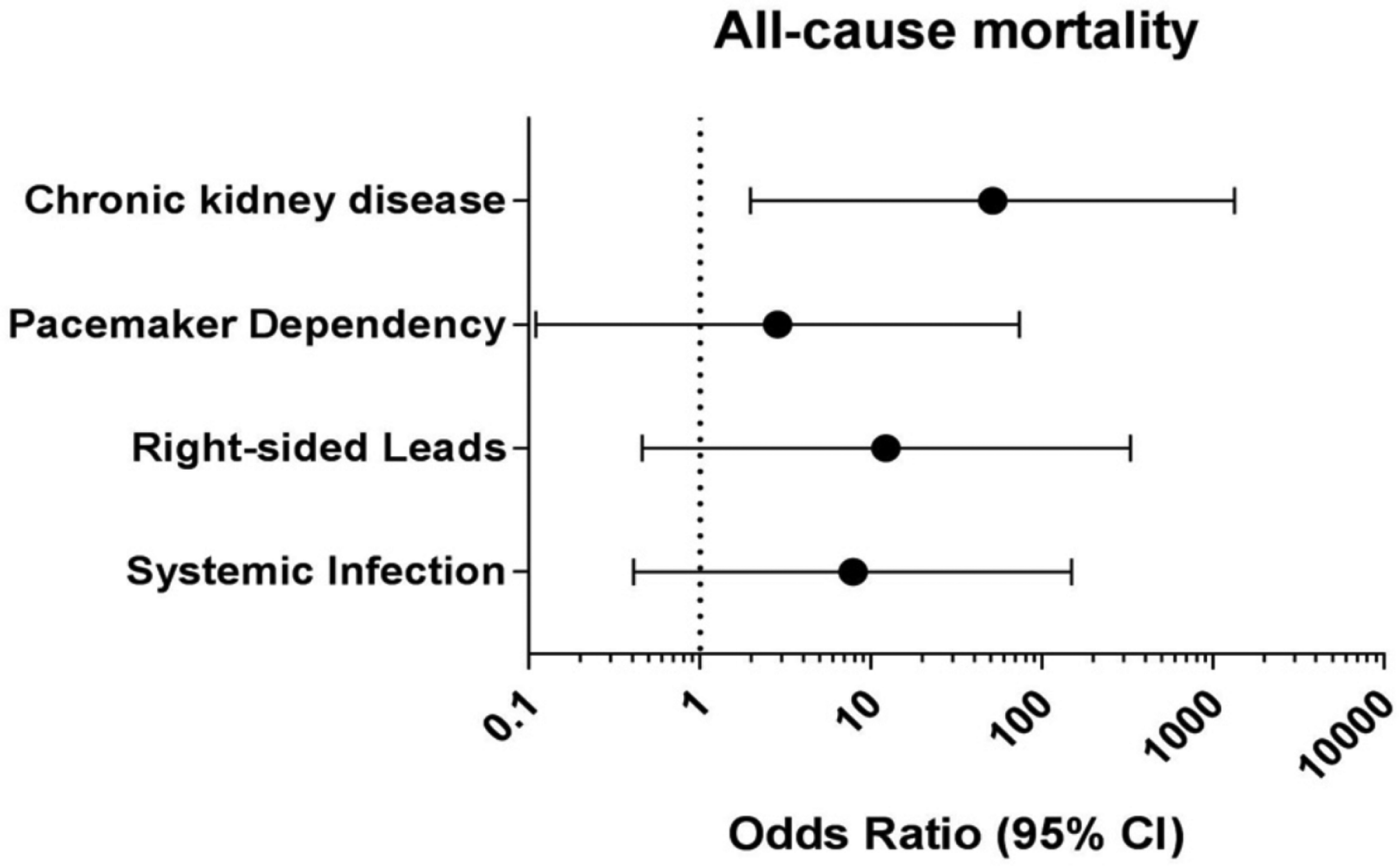
Multivariable logistic regression analysis to identify independent predictors for all-cause mortality in young patients undergoing TLE. CI: Confidence interval.

**Figure 3:**
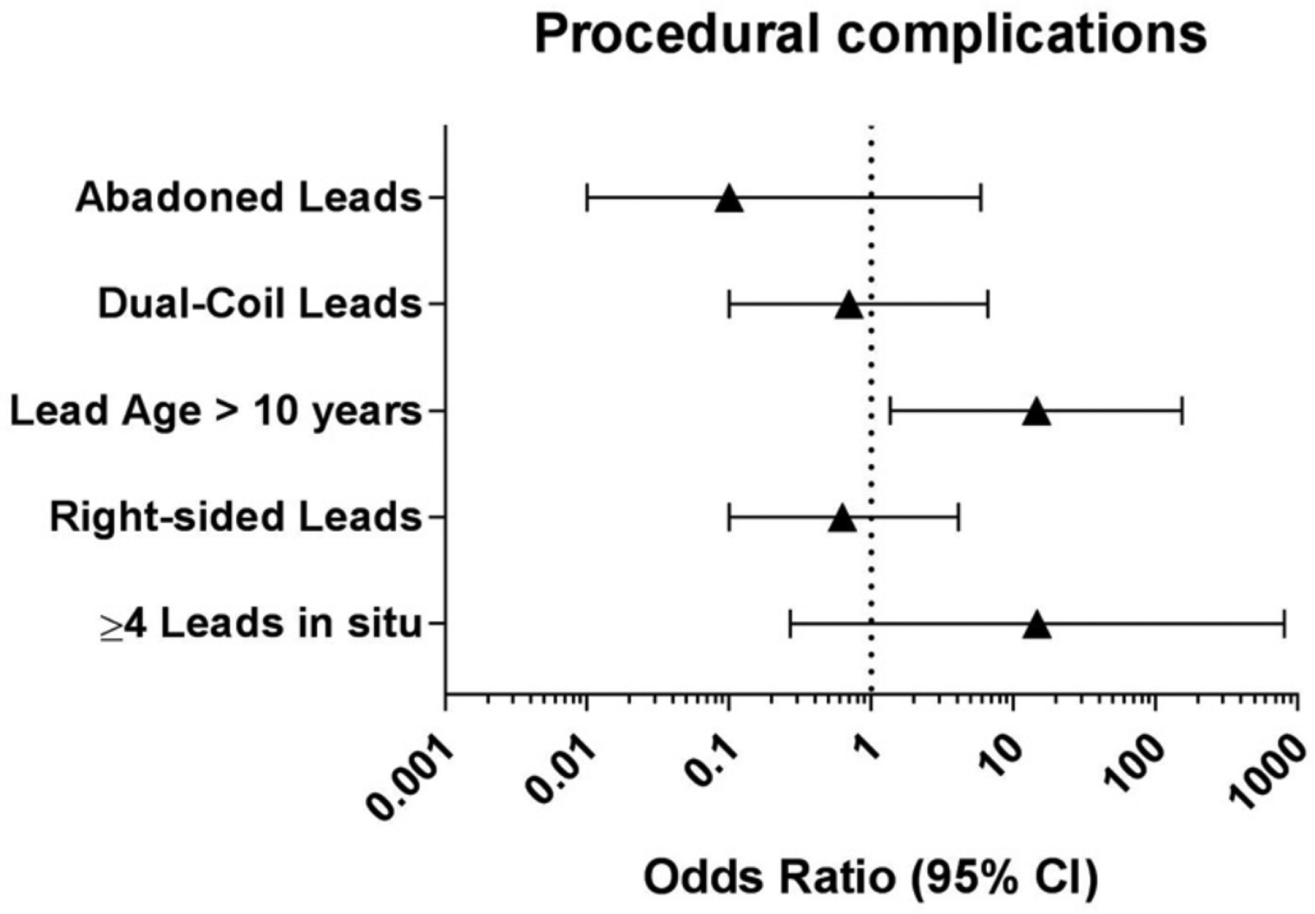
Multivariable logistic regression analysis to identify independent predictors for procedural complications in young patients undergoing TLE. CI: confidence interval.

Predictors of adverse outcomes, such as procedural complications and all-cause mortality were analyzed by uni- and multivariable logistic regression. Univariable analysis revealed CKD (OR: 25.0; 95% CI: 2.99–208.9; *p* = 0.003) and systemic infection (OR: 15.7; 95% CI: 1.57–157.3; *p* = 0.019) as predictors of all-cause mortality. After adjustment in multivariable analysis, CKD (OR: 19.0; 95% CI: 1.84–194.9; *p* = 0.018) and systemic infection (OR: 12.7; 95% CI: 1.14–142.8; *p* = 0.039) remained predictive for all-cause mortality in our subgroup (Table 4 and Figure 2). Lead age ≥ 10 years (OR: 14.58, 95% CI: 1.36–156.2; *p* = 0.027) was identified as sole independent risk factor for procedural complications after multivariable analysis (Table 5 and Figure 3).

**Table 4:** Uni- and multivariable regression analysis for all-cause mortality in patients aged ≤ 45 years undergoing TLE.

Variables	Univariate analysis			Multivariate analysis		
	OR	95% CI	*p*-value	OR	95% CI	*p*-value
Abandoned leads	0.88	0.09–8.65	0.910			
Chronic kidney disease	25.0	2.99–208.9	0.003	19.0	1.84–194.9	0.018
Dual-coil leads	2.81	0.38–20.6	0.310			
Lead age* *≥* *10 years	1.04	0.09–11.8	0.974			
LVEF* *<* *30%	2.73	0.27–27.7	0.397			
Pacemaker dependency	7.17	0.73–70.8	0.092			
Right-sided leads	8.42	0.85–83.2	0.068			
Systemic infection	15.7	1.57–157.3	0.019	12.7	1.14–142.8	0.039
≥4 leads *in situ*	2.56	0.25–25.9	0.427			

*P*-value < 0.05 was considered statistically significant; BMI, body mass index; CI, confidence interval; LVEF, left ventricular ejection fraction; OR, odds ratio.

**Table 5:** Multivariable logistic regression analysis to identify independent predictors for procedural complications in patients aged ≤ 45 years undergoing TLE.

Variables	Odds ratio	95% Confidence interval	*p*-value
Abandoned leads	0.1	0.01–5.92	0.264
Dual-coil leads	0.70	0.10–6.65	0.754
Lead age* *≥* *10 years	14.58	1.36–156.2	0.027
Right-sided leads	0.63	0.10–4.12	0.625
≥4 leads *in situ*	14.72	0.27–811.1	0.189

*P*-value < 0.05 was considered statistically significant.

## 4. Discussion

To the best of our knowledge, this study represents the largest subgroup analysis conducted to date, focusing specifically on the outcome of young patients undergoing lead extraction using laser sheaths. The retrospective analysis from El-Chami et al. (19) included the data of 84 patients under the age of 40 years extracted with LLE, whereas in our subgroup analysis of the multicenter GALLERY-study (6) 160 patient with an age of 45 years or younger were identified.

In the GALLERY a total number of extracted leads was 6,117, of which 5.6% (348/6,117) were implanted in young patients.

The main findings in our analysis are the following:

1. Lead dysfunction was the main indication for lead extraction in young patients (*p* < 0.001).
2. The median procedure time was significantly higher in the young patients (*p* = 0.02)
3. Complications rate, procedural- and clinical success, procedural-related and all-cause mortality were similar in both groups.
4. CKD and systemic infection were identified as independent predictors of all-cause mortality, while lead age ≥10 years was found to be the sole independent risk factor for procedural complications.
5. Procedure-related mortality in the young population was 0.0% and the all-cause mortality (2.5%) was primarily driven by septic shock (1.9%).

The finding that lead dysfunction was the primary indication for lead extraction in our study population aligns with the data from other studies that have analyzed young patient cohorts (15, 19–21). This consistency across studies suggests that lead dysfunction is a common and significant issue leading to the need for lead extraction in younger patients, which is mainly caused by the somatic growth, mechanical stress and higher tissue proliferation (16, 17). In addition, different studies (20, 22) have demonstrated that a younger age (<8 years or <12 years) at implantation has an incremental risk for lead failures. The data of the exact age at implantation and data regarding recalled leads such as Riata (Abbott, St. Jude Medical) or Sprint fidelis (Medtronic) are missing in our database. Kleemann et al. (23) showed that patients with lead defects are younger, more often female, and have better preserved LVEF. This applies also to our study cohort, which had a better LVEF, and female sex was significantly more often represented than in the older group.

Another finding was that the median procedure time was significantly longer in the young patients, though the mean number of total leads was significantly higher in the older population. Also lead age and the number of abandoned leads was similar in both groups with a higher number of ICD-leads extracted in the young patients (Table 2). An explanation for the longer procedure time could be the higher degree of adhesions and fibrosis described in the young population (17). Segreti et al. (24) studied the major predictors of fibrous adherence in ICD-leads in comparison to PM-leads and found similar time-depended fibrous formation in both lead types, but greater distribution of fibrous tissue along the course of the ICD-leads. Furthermore, they showed that dual-coil technology and passive fixation mechanism are also predictors for adherence. In our study there were no significant difference regarding the presence of dual-coil leads between the groups. The older population had a significantly higher number of passive fixation than the younger one. But the data of fixation mechanism was incomplete so that no clear statement can be made in this regard. Kutarski et al. (15) also described a longer mean extraction time per lead in young adults, which they attributed to the need to use alternative approaches and second line tools in this cohort. There was no significant difference in the utilization of additional tools in our study, however. Furthermore, El Chami et al. (19) described a rate of 10% for the need of femoral access in their young population showing the challenging nature of TLE in this patient.

Based on the data of the studies mentioned above (15, 17, 19, 24), it can be inferred that factors such as fibrous adherence, the use of additional tools, and the need for additional femoral access may contribute to longer procedure times in the young patient population undergoing LLE. While the specific information regarding the amount of femoral access needed is missing in our database, the data from the referenced studies (15, 17, 19, 24) provide some insights into the potential factors influencing procedure duration in this population. These factors can lead to increased complexity and challenges during the extraction procedure, which in turn may contribute to longer procedure times.

Large registry studies such as LExICon, ELECTRa and GALLERY proved the safety and efficacy of LLE. The rates for complications, procedural success, clinical success, procedural-related mortality, and all-cause mortality were similar between the two age groups in our study. While Burger et al. (25) demonstrated the safety and feasibility LLE in the octogenarian population, our analysis indicates that LLE is a feasible and safe procedure also in the young population. In contrast, a subgroup analysis of the PROMET-Study (26), which focused on mechanical lead extraction with MDR sheaths, showed a numerically lower rate of procedural success in younger patients compared to the older population. This was attributed to a higher prevalence of fibrous encapsulation and a higher number of ICDs in the young cohort. Interestingly, in our study, despite the higher prevalence of ICDs in the young population, the rate of procedural success was similar between both age groups. This highlights the feasibility and efficacy of LLE in young patients.

According to the retrospective analysis by Diaz et al. (27), there was an increased mortality associated with the use of laser sheath extraction tools compared to mechanical tools. On the other hand, the recent study by Zsigmond et al. (28), which conducted a head-to-head comparison of laser versus powered mechanical sheaths as first-choice and second-line extraction tools, found no significant difference in major or minor complications between the two approaches. However, the study did observe a significant increase in clinical success rate when mechanical tools were used as a second-line option. In our study the laser sheath was use as a first choice due to its unique properties, specifically its flexibility and ability to adapt to venous anatomy, especially in challenging areas such as the confluence of the brachiocephalic veins into the superior vena cava (SVC). Additionally, the energy level can be adjusted, which is beneficial in cases with complicated adhesions, helping to minimize the risk of vascular tears and other complications.

In terms of procedural-related complications, our results indicated that lead dwell time exceeding 10 years was a significant predictor. This finding aligns with the results of the ELECTRa study, where lead dwell time over 10 years was also associated with a higher risk of procedural complications and failure. Furthermore, in the subgroup analysis of the GALLERY study focusing on patients undergoing TLE for CDRI, Chung et al. (8) similarly found that lead dwell time exceeding 10 years was a predictor for both procedural failure and complications. These findings highlight the importance of considering these risk factors when evaluating the feasibility and potential risks of lead extraction procedures.

In our subgroup analysis, CKD was identified as an independent predictor for all-cause mortality in the young population undergoing TLE. This finding is consistent with previous studies that have shown an increased mortality risk in CKD patients after TLE (6, 8, 13, 18). A meta-analysis conducted by Tan et al. (29) compared the risk of mortality in CKD patients versus control patients after TLE. The results showed a significantly higher all-cause mortality risk in CKD patients compared to the control group. The increased mortality risk was evident both in the short-term (follow-up time ≤1 year) and long-term (follow-up time >1 year). The hazard ratio (HR) for all-cause mortality was approximately 1.99 at follow-up ≤1 year and 2.36 at follow-up >1 year for CKD patients.

Despite the low event rate, our analyses demonstrated that systemic infection is a predictor for all-cause mortality also in the young patients. It is noteworthy that despite the significantly lower prevalence of comorbidities in the young population (Table 1), CDRI can still lead to fatal outcomes. While there are clear recommendations (9, 10) for lead extraction in patients with CDRI, opinions regarding lead extractions for non-infectious indications remain controversial (30–33). The lack of clear recommendations in the young population, where lead fracture is the main indication, is a concern. Certain risk factors, such as lead burden exceeding two leads, generator replacement, and device upgrades, increase the likelihood of CDRI (34), which correlates with higher rates of all-cause in-hospital mortality and procedure-related mortality (8). On the other hand, lead extraction has been associated with lower adjusted 5-year infection rates compared to cap and abandoned leads (35). Furthermore, Elgaard et al. (30) demonstrated in their long-term follow-up study of abandoned ICD leads, that the probability of lead extraction increased to 5.5%, 7.6%, and 15.2% after 2.5, 5, and 10 years of abandonment, respectively. In this content, it is important to highlight that in our study, no procedure-related mortality (0.0%) occurred in the young population and the all-cause mortality (2.5%) was primarily associated with septic shock (1.9%). These results emphasize the safety of LLE in this age group. When combined with the previously mentioned findings, these results offer additional support for advocating the use of lead extraction in the ongoing debate. However, despite the low occurrence of procedural complications, it is crucial to perform a careful and individualized evaluation, considering factors that may pose potential risks. In such cases, risk assessment tools like the ELECTRA Registry Outcome Score (EROS) (36) can be helpful in identifying patients who are at a higher risk of significant procedural complications, aiding in the decision-making process and refer these patients in high volume centers.

## 5. Conclusion

TLE in young patients is safe and effective with a procedure-related mortality rate of 0.0%. CKD and systemic infection are predictors for all-cause mortality, whereas lead age ≥ 10 years was identified as independent risk factor for procedural complications in young patients undergoing TLE.

## 6. Limitations

The retrospective nature of our study poses a potential limitation, as it may introduce selection bias and detection bias. Despite efforts to minimize biases using standardized definitions and a predefined case report form, the influence of individual physician decisions and variations in therapeutic approaches cannot be completely eliminated. The lack of data on implantation indication and the use of alternative access methods limits a comprehensive understanding of these factors’ impact on the outcomes. Additionally, low event rates for adverse events in our subgroup may have led to less pronounced differences in inter-group comparisons and may have dimished the impact of certain predictors for regression analyses. Finally, the inclusion of only high-volume referral centers with a specific expertise in TLE may result in a skewed representation of patient characteristics and procedural outcomes, limiting the generalizability of the findings to other settings. It is important to interpret the results with caution and consider the specific context in which the study was conducted.

## Data Availability

The data analyzed for the purpose of the study are included in the article, the original data can be provided upon request to the corresponding author.
